# Circulating tumor DNA predicts recurrence and assesses prognosis in operable gastric cancer: A systematic review and meta-analysis

**DOI:** 10.1097/MD.0000000000036228

**Published:** 2023-12-01

**Authors:** Junjie Mi, Rong Wang, Xiaofang Han, Ruijun Ma, Huiying Li

**Affiliations:** a Department of Gastroenterology, Shanxi Provincial People’s Hospital (The Fifth Hospital of Shanxi Medical University), Taiyuan, China; b Core Laboratory, Shanxi Provincial People’s Hospital (The Fifth Hospital of Shanxi Medical University), Taiyuan, China; c Fenyang College of Shanxi Medical University, Fenyang, China.

**Keywords:** circulating tumor DNA, gastric cancer, meta-analysis, prognosis, recurrence

## Abstract

**Background:**

Selecting the appropriate patient for further treatment after surgery for gastric cancer can improve the patient prognosis. Circulating tumor DNA (ctDNA) has the potential to predict recurrence and prognosis after gastric cancer surgery, but the results are still inconclusive. As the completed studies had small sample sizes and were inconsistent, a meta-analysis was conducted to assess the effect of ctDNA on recurrence and prognosis after gastric cancer surgery.

**Methods:**

PubMed, Embase, Scopus, and the Web of Science were searched for potentially eligible studies published up to April 7, 2023. Pooled relative risk (RR) and pooled hazard ratio (HR) were calculated to evaluate recurrence, recurrence-free survival (RFS), and overall survival (OS) following gastric cancer surgery.

**Results:**

A pooled analysis revealed that patients who were ctDNA positive before and after surgery were at a high risk of gastric cancer recurrence (RR = 1.79, 95% CI: 1.19–2.71; RR = 3.17, 95% CI: 2.36–4.25). The pooled data revealed that ctDNA-positive patients had a poorer RFS and OS (HR = 6.37, 95% CI: 2.70–15.01; HR = 4.58, 95% CI: 1.68–12.49).

**Conclusions:**

ctDNA-positive patients were at a high risk of recurrence after gastric cancer surgery and had a poorer prognosis. Hence, ctDNA-positive patients needed close follow-up and further treatment.

## 1. Introduction

Gastric cancer has the fifth-highest incidence rate and mortality rate in the world, causing 782,685 cancer deaths each year and posing a serious health risk.^[1]^ Despite the rapid development of new treatments, perioperative chemotherapy and radical surgery remain the mainstays of localized gastric cancer treatment.^[2]^ Despite localized gastric cancer undergoing curative gastrectomy, the recurrence rate of gastric cancer was 43% at 2 years and up to 85% at 5 years after surgery, and most gastric cancer recurrences were already in the progressive stage with no chance of cure.^[3]^ Only 3.2% of patients with recurrent gastric cancer received potentially curative treatment.^[4]^ Molecular residual disease (MRD) is the main cause of the recurrence of gastric cancer after surgery. The sensitivity of clinical imaging and tumor markers for postoperative MRD in gastric cancer was low, with carcinoembryonic antigen and carbohydrate antigen 19-9 having a sensitivity of only 34% and 24%, respectively, in diagnosing postoperative recurrence of gastric cancer.^[5]^ In addition, contrast-enhanced computed tomography (CT) was the most commonly used imaging method to detect recurrence of gastric cancer, with a sensitivity of only 70%–73%.^[6]^ Therefore, there is an urgent need for highly accurate biomarkers to detect recurrence of gastric cancer in clinical practice so that patients with recurrence can receive timely treatment and ultimately improve their survival.^[7,8]^

Circulating tumor DNA (ctDNA) is a fragment of DNA that is released into the bloodstream when a tumor undergoes necrosis or apoptosis.^[9]^ ctDNA contains complete information on tumor variation and has developed rapidly in recent years for guiding tumor treatment and monitoring treatment outcome and prognosis.^[10–12]^ The concentration of ctDNA in the blood is very low and accounts for approximately 0.01%–2% of cell-free DNA (cfDNA), where cfDNA is derived from white blood cells and consists of double-stranded DNA fragments of approximately 140 to 170 base pairs (bp) in length and ctDNA usually consists of < 145 bp in length. Moreover, previous biotechnologies had low sensitivity for detecting ctDNA.^[13,14]^ The sensitivity of ctDNA testing has gradually improved with the development of molecular biology techniques, particularly next-generation sequencing and droplet digital PCR and cancer personalized profiling by deep sequencing and tagged-amplicon deep sequencing, etc.^[9,15]^ ctDNA as part of a liquid biopsy has the advantage of being noninvasive and easily sampled multiple times. Additionally, ctDNA can detect tumor variation even when tumor tissue is not available and can avoid tumor tissue heterogeneity in biopsies.^[16]^ ctDNA has been shown to detect the recurrence of breast, lung, colon, and bladder cancers after surgery.^[17–21]^ Although several studies have explored ctDNA as a predictor of recurrence of gastric cancer after surgery in recent years, the current studies suffer from small sample sizes and inconsistent or even opposite results.^[22–29]^ We will use meta-analysis to explore ctDNA ability to predict recurrence of gastric cancer after surgery and provide new evidence for the clinical application of ctDNA in gastric cancer. The primary aim of the study was to assess ctDNA to predict the recurrence of operable gastric cancer. A secondary aim was to use ctDNA to assess the prognosis of gastric cancer.

## 2. Methods

### 2.1. Literature search

This meta-analysis was carried out according to the preferred reporting items for systematic reviews and meta-analyses guidelines and the meta-analysis of observational studies in epidemiology.^[30,31]^ See Supplementary Appendices 1, http://links.lww.com/MD/K793 and 2, http://links.lww.com/MD/K794. With a search deadline of April 7, 2023, we searched PubMed, Embase, Scopus, and Web of Science for studies on ctDNA predicting recurrence risk and assessing prognosis in operable gastric cancer patients. Supplementary Appendix 3, http://links.lww.com/MD/K795 provides a detailed procedure. Based on the inclusion and exclusion criteria, 2 investigators (Mi, Li) independently screened studies from the database, extracted and entered relevant eligible studies, and in the event of disagreement, the final outcome was determined by adding a third person (Wang) to the discussion. Papers that were plainly irrelevant to the study were excluded by reading the title and abstract in accordance with the inclusion and exclusion criteria, and the full text of the remaining papers was read to determine if they were eventually included. The process of retrieval in this study was not limited by language. The study search technique included a combination of subject terms and free words, and it was as follows: (Minimal residual disease OR Recurrence OR Prognosis OR MRD OR Neoplasm recurrence) AND (Gastric Cancer OR Stomach Neoplasms OR Stomach cancer OR Stomach tumor OR Gastric tumor) AND (“Tumor DNA AND Cell-Free” OR “Cell-Free Tumor AND DNA” OR Cell Free Tumor DNA OR “Tumor DNA AND Circulating” OR “Circulating Tumor AND DNA” OR ctDNA OR Circulating Tumor DNA). In addition, to avoid omissions, the investigators looked for references in the included study.

### 2.2. Selection and exclusion criteria

The inclusion criteria were as follows: patients were diagnosed with operable gastric cancer; investigation of the role of ctDNA in the prediction of tumor recurrence risk and assessment of prognosis; studies that provided enough information to calculate the RR for predicting tumor recurrence and HR for assessment of prognosis; and human clinical trial studies. The following were the exclusion criteria: meta-analysis, reviews, and letters; studies for which relevant raw data were not available; studies involving co-morbidities with other malignant diseases; studies involving gastrooesophageal cancer; and studies detecting cfDNA.

### 2.3. Data extraction and quality assessment

Two investigators (Han, Wang) independently collected data for each included study by developing a data extraction form, and if the comparison of the extracted data did not agree, the disagreement was resolved by consulting a third person (Ma). For the risk of tumor recurrence, the number of ctDNA-positive patients and the number of ctDNA-negative patients were extracted. Regarding the prognostic indicators overall survival (OS) and recurrence-free survival (RFS), hazard ratio (HR) as well as 95% confidence intervals (95% CI) were extracted for each study. The setting of ctDNA positivity thresholds for each included study was derived from the original study. If the relevant data is not available from the original study, we will contact the corresponding author of the original study to obtain the raw data.

The data extract also included the following basic information: first author, year of publication, country, patient count, average age, gender, number of serum samples, sample type, testing methods, number of genes, number of centers, study design, TNM staging, clinical stage, median follow-up, ctDNA testing time, definition of the positive ctDNA, and treatment strategy. In addition, data related to ctDNA shedding for different types of gastric cancer were also extracted. We used the Newcastle-Ottawa scale to rate the included studies from 0 to 9 based on the content of the included studies and the scale judgement criteria. A total of 20 entries in the Reporting Recommendations for Tumor Marker Prognostic Studies list contain an introduction, materials, methods, results, and discussion that were used to assess the quality of the included studies.

### 2.4. Statistical analysis

The effect sizes for risk of tumor recurrence were assessed by the Mantel Haenszel method as relative risk (RR) and 95% CI, and for prognosis as HR and their 95% CI for OS and RFS. To gain a thorough understanding of ctDNA role in predicting the prognosis of gastric cancer, we evaluated the baseline preoperative and postoperative RFS and OS. To assess the extent of heterogeneity among the included studies, we utilized a Chi-squared Q test and I^2^ coefficients. A statistically significant difference was considered at *P* < .05, employing a random effects model. In addition, we performed sensitivity analyses to test the stability of the meta-analysis results, and finally, the egger test and harbor test were used to assess whether there was publication bias in this study. The overall effect test was statistically significant at *P* < .05, and STATA 17.0 software was used to conduct all analyses.

## 3. Results

### 3.1. Search results

**Figure 1.**
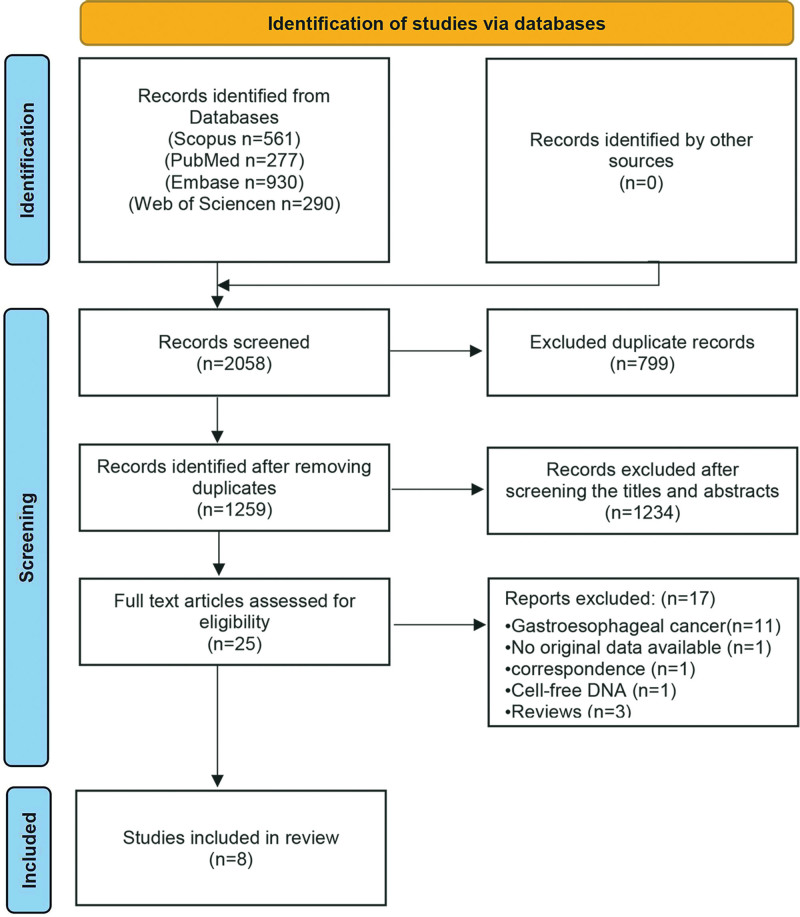
Literature search and study selection flow diagram for systematic reviews.

An initial selection of 2058 articles was achieved after careful searching of the databases according to the search strategy that had been developed. After 799 duplicate articles were removed, 1234 irrelevant articles were excluded after careful reading of the titles and abstracts. After conducting a thorough in-depth review of the full-text articles, we excluded 17 studies. These exclusions encompassed articles related to gastroesophageal cancer, correspondence, cfDNA, reviews, and studies lacking original data. The final meta-analysis included 8 studies with a total of 423 patients, as detailed in Figure 1.

### 3.2. Characteristics of eligible studies and quality assessment

A total of 423 patients and 884 serum samples were included in the meta-analysis of 8 studies published between 2019 and 2022, in which the study characteristics and demographics are summarized in Table 1. The timing of ctDNA testing, the definition of positive ctDNA, and treatment strategies were summarized in Supplementary Appendix 4, http://links.lww.com/MD/K796. Supplementary Appendix 5, http://links.lww.com/MD/K797 provided a comparison of ctDNA shedding according to gastric cancer type, different stages, localization, pathological response, and degree of differentiation. The results of the assessment of the quality of the included studies by means of the Newcastle-Ottawa scale, which includes 3 subsets of selection comparability and outcome, are shown in Supplementary Appendix 6, http://links.lww.com/MD/K798. The quality assessment of the included studies showed that the included studies were of high quality, which was also confirmed by the Reporting Recommendations for Tumor Marker Prognostic Studies checklist in Supplementary Appendix 7, http://links.lww.com/MD/K799.

**Table 1:** Characteristics of the included studies in this meta-analysis.

First author	Kim	Cabel	Yang	Leal	Suzuki	Fedyanin	Huffman	Yuan
Publication (yr)	2019	2019	2020	2020	2020	2020	2022	2022
Country	Korea	France	China	USA	Japan	Russia	USA	China
Patients (n)	19	19	46	50	46	42	88	100
Mean age (range/IQR)	60 (41–77)	62 (45–78)	54 (28–78)	60 (30–77)	72 (46–92)	NA	NA	NA
Male (%)	88	84	83	72	76	NA	NA	NA
Serum samples (n)	107	50	296	120	40	NA	271	NA
Sample type	Plasma	Plasma	Plasma	Plasma	Plasma	Plasma	Plasma	Plasma
Tissue testing methods	WGS	NGS	NGS	NA	NA	NGS	WES	NGS
ctDNA testing methods	PCR	ddPCR	NGS	NGS	ddPCR	ddPCR	NGS	NA
Tissue tumor oncogenes (n)	NA	39	1021	NA	NA	50	NA	425
ctDNA oncogenes (n)	NA	5	50	58	52	NA	NA	NA
Post-operative ctDNA testing time	< 12 mo	<1 mo	9–48 d	6.5 wk	7.3 d	7 d	16 wk	4 d
The time ahead for clinical detection	4.05 mo	NA	6 mo	8.9 mo	6 mo	NA	NA	NA
Centers (n)	Multicentre	Single center	Single center	Multicentre	Multicentre	NA	Multicentre	NA
Study design	Retrospectively	Prospectively	Prospectively	Retrospectively	Prospectively	Prospectively	Retrospectively	Prospectively
Clinicalstage	II-IV	NA	I-III	IA-IV	II-III	I-IV	I-III	II-III
TNM staging	T3-T4b/N0-N3b/M0	T1-T4/N0-N+	pT1a-pT4a/pN0-pN3b/pM0	ypT0-ypT4b/ypN0-ypN3b/ypM0-ypM1	NA	NA	NA	NA
Median follow-up (range/IQR, mo)	12	26 (11–35)	29.1 (5.7–32.3)	42	12.2	NA	34.8	NA

dd PCR = droplet digital polymerase chain reaction, IQR = interquartile range, n = number, NA = not available, NGS = next-generation sequencing, PCR = polymerase chain reaction, WES = whole-exome sequencing, WGS = whole-genome sequencing.

### 3.3. Preoperative ctDNA predicts the risk of gastric cancer recurrence

**Figure 2.**
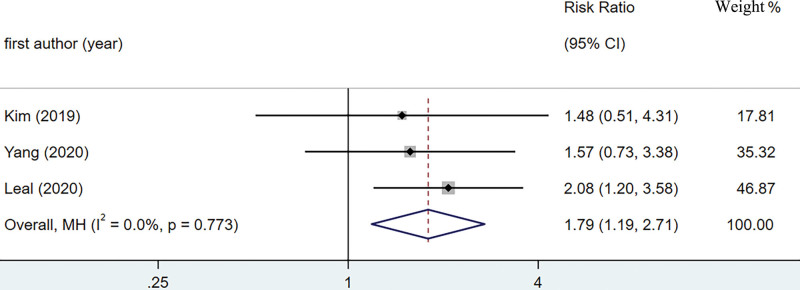
A forest plot of the pooled effect for relative risk in presurgical ctDNA prediction of gastric cancer recurrence after surgery. ctDNA = circulating tumor DNA.

A total of 3 studies were included in the pooled analysis of preoperative ctDNA to predict the risk of recurrence of gastric cancer.^[22–24]^ The heterogeneity of preoperative ctDNA predicting postoperative recurrence of gastric cancer was small, so we used a fixed effects model to calculate the combined RR as shown in Figure 2 (RR = 1.79, 95% CI: 1.19–2.71, *P* < .05; I^2^ = 0.0%, *P* > .05). Preoperative ctDNA positivity was associated with a higher risk of gastric cancer recurrence in postoperative patients with gastric cancer. The 3 studies included in the pooled analysis showed inconsistent results, with Kim study and Yang study showing that although there was a tendency for preoperative ctDNA positivity to be associated with greater tumor recurrence, the results did not reach statistical significance, possibly due to the small sample sizes included in these 2 studies.^[22,24]^ However, Leal study showed that pre-operative ctDNA positive patients have a higher risk of recurrence of gastric cancer (RR = 2.08, 95% CI: 1.20–3.58).^[23]^

### 3.4. Postoperative ctDNA predicts the risk of gastric cancer recurrence after surgery

**Figure 3.**
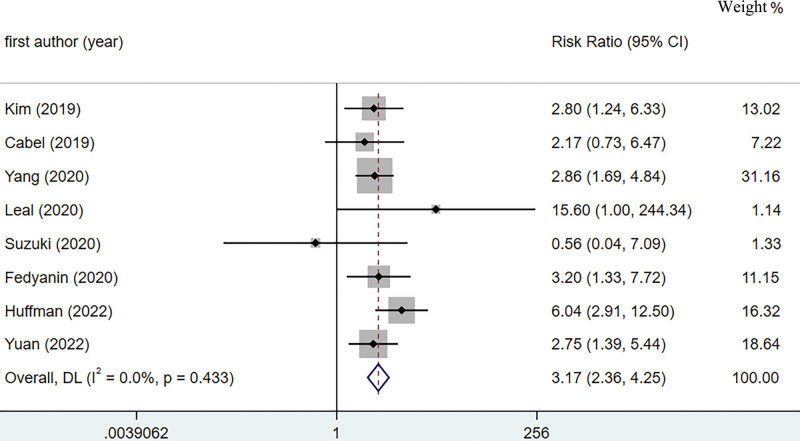
A forest plot of the pooled effect for relative risk in the prediction of gastric cancer recurrence after surgery using post-surgical ctDNA. ctDNA = circulating tumor DNA.

A total of 8 studies were included in the pooled analysis of postoperative ctDNA to predict the risk of tumor recurrence after surgery.^[22–29]^ To fully explore the postoperative ctDNA prediction of postoperative tumor recurrence, we used a random-effects model and a fixed-effects model to calculate the combined RR as shown in Figure 3 and Supplementary Appendix 8, http://links.lww.com/MD/K800, respectively. Postoperative ctDNA-positive patients had a higher risk of tumor recurrence, with a pooled RR of 3.17, 95% CI:2.36–4.25 and 3.68, 95% CI:2.66–5.09) for both the random-effects and fixed-effects models, respectively. Although both statistical models showed that postoperative ctDNA was a good predictor of postoperative gastric cancer recurrence, the results of the random-effects model were more conservative and reliable than the fixed-effects model. The 8 studies included in the pooled analysis showed inconsistent results, with the Cabel and Suzuki studies showing that postoperative ctDNA positivity did not predict postoperative tumor recurrence, possibly due to the small sample sizes included in these 2 studies.

### 3.5. Prediction of RFS by ctDNA

**Figure 4.**
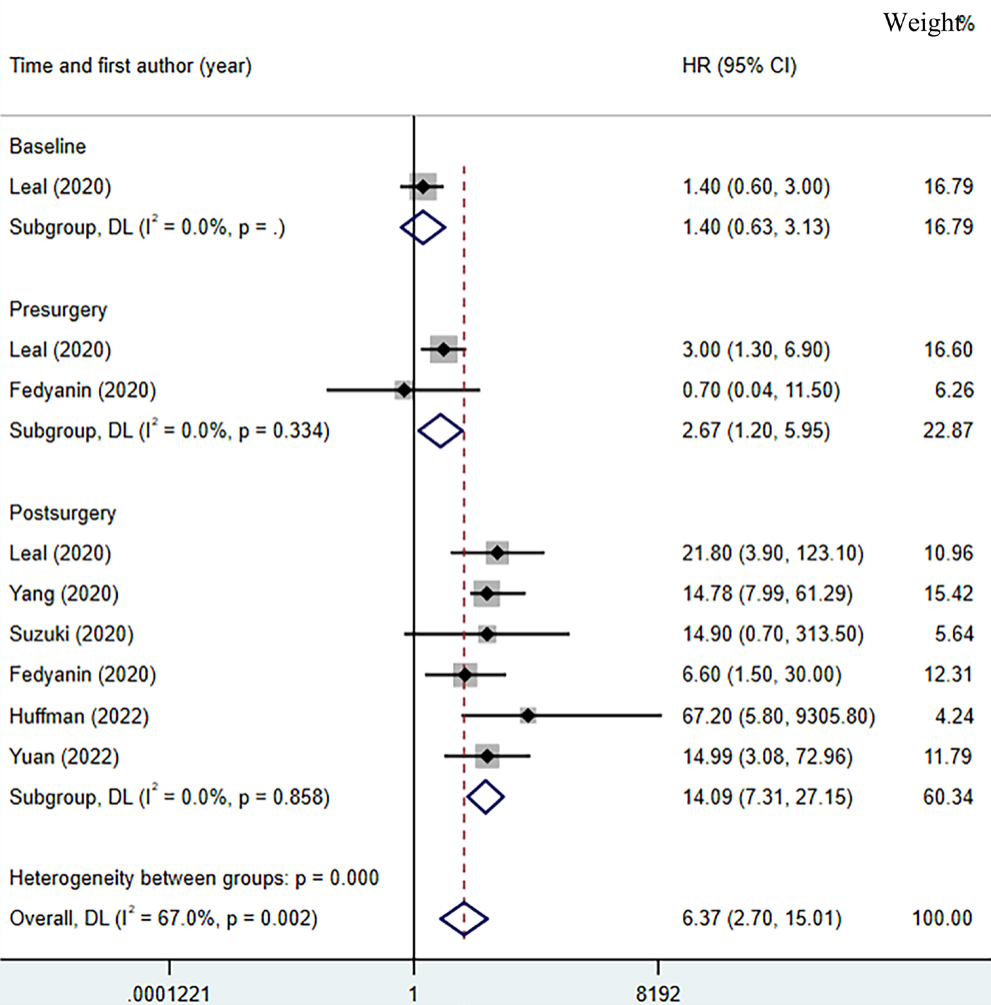
Forest plot on ctDNA prediction of recurrence-free survival with a pooled effect hazard ratio. ctDNA = circulating tumor DNA.

A total of 6 studies were included in the pooled analysis of ctDNA for predicting postoperative RFS in gastric cancer.^[22,23,26–29]^ In the pooled analysis, Leal study and Fedyanin study were included separately for different time points of ctDNA testing.^[23,29]^ The heterogeneity of ctDNA in predicting postoperative RFS in gastric cancer was large, so we used a random effects model to calculate the pooled HR in Figure 4 (HR = 6.37, 95% CI: 2.70–15.01, *P* < .05; I^2^ = 67%, *P* < .05). The high heterogeneity may be due to the different methods used to detect ctDNA and the different demographics of the included studies. Regarding prognosis after surgery for gastric cancer ctDNA-positive patients have worse RFS. Only one study of ctDNA testing at baseline showed that baseline testing of ctDNA was not associated with RFS after gastric cancer surgery.^[23]^ However, pooled results showed that preoperative ctDNA-positive patients had a worse RFS (HR = 2.67, 95% CI: 1.20–5.95).^[23,29]^ Although postoperative ctDNA yielded inconclusive results in predicting RFS, a pooled analysis revealed that postoperative ctDNA positive patients had a worse RFS (HR = 14.09, 95% CI: 7.31–27.15).

### 3.6. Prediction of OS by ctDNA

**Figure 5.**
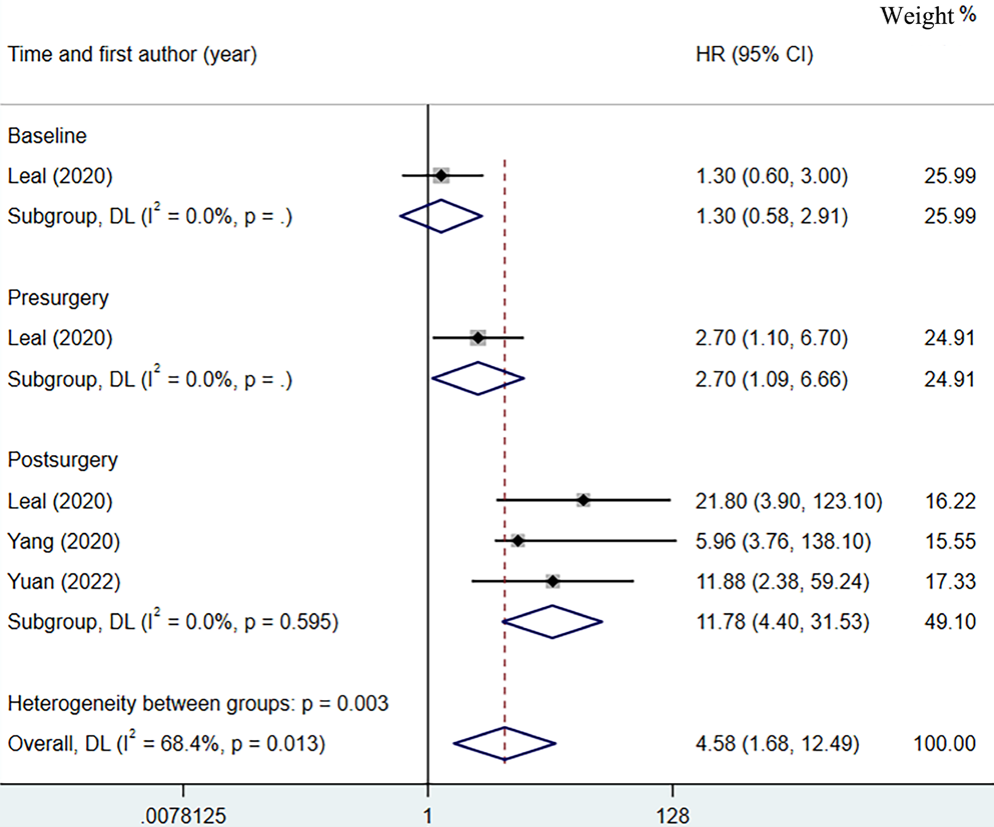
Forest plot on ctDNA prediction of overall survival with a pooled effect hazard ratio. ctDNA = circulating tumor DNA.

A total of 3 studies were included in the pooled analysis of ctDNA predicting OS in patients with postoperative gastric cancer.^[22,23,27]^ The different time points for ctDNA testing in Leal study were included separately in the pooled analysis. The heterogeneity of ctDNA in predicting postoperative OS in gastric cancer was large, so we used a random effects model to calculate the pooled HR in Figure 5. The high heterogeneity may be due to the different methods used to detect ctDNA and the different follow-up times. Pooled analysis showed ctDNA-positive patients had worse OS (HR = 4.58, 95%CI: 1.68–12.49, *P* < .05; I^2^ = 68.4%, *P* < .05). Leald study showed that baseline ctDNA testing was not associated with OS after surgery for gastric cancer, but preoperative ctDNA positive patients had worse OS (HR = 2.7 95% CI: 1.09–6.66). Additional pooled analysis showed that post-operative ctDNA positive patients had worse OS (HR = 11.78, 95% CI: 4.40–31.53).

### 3.7. Publication bias and sensitivity analysis

The egger test and harbor test were used to assess publication bias, and the results showed no significant publication bias (*P* = .876 and *P* = .426) in Supplementary appendix 9, http://links.lww.com/MD/K801 and Supplementary appendix 10, http://links.lww.com/MD/K802. Sensitivity analyses were used to examine the effect of individual studies on the stability of the pooled ctDNA for predicting the risk of tumor recurrence, and the pooled RRs and 95% CIs after sequential removal of each study indicated that none of the studies could significantly affect the pooled effect (see Supplementary Appendix 11, http://links.lww.com/MD/K803).

## 4. Discussion

Early detection of postoperative recurrence in patients with gastric cancer can facilitate timely adjustment of treatment plans and have a positive impact on improving the prognosis of patients. For those at high risk of tumor recurrence, multimodal treatment, including radiotherapy, and individualized follow-up should be undertaken. Most recurrences of gastric cancer are diagnosed in the advanced stage, and the lack of effective treatment in the advanced stage contributes to the high mortality rate of gastric cancer.^[32]^ In addition, despite the use of multimodal treatment for gastric cancer, the recurrence rate after surgery is high, and most gastric cancer patients experience local recurrence, peritoneal metastases, and distant metastases after surgery.^[33,34]^ The risk of recurrence after surgery for gastric cancer is now mainly assessed by the pathological staging and tumor regression grading system, but its application in clinical practice is somewhat limited.^[4,35,36]^ In addition, the sensitivity of current imaging and tumor markers for monitoring recurrence of gastric cancer is insufficient, so there is an urgent clinical need for a biomarker that can accurately monitor recurrence of gastric cancer after surgery. ctDNA derived from necrotic or apoptotic tumor cell DNA fragments with oncogene variants shows promise in predicting tumor recurrence after surgery. This study showed that pre- and postoperatively positive ctDNA patients had a higher risk of postoperative recurrence of gastric cancer (RR = 1.79, RR = 3.17), as well as a worse FRS (HR = 6.37) and worse OS (HR = 4.58).

Gastric cancer is a very heterogeneous malignancy, and studies have shown that not all types of gastric cancer can detect ctDNA shedding; the current preoperative detection rate of ctDNA is about 30%–50%.^[37–40]^ For ctDNA non-shedders, ctDNA remained negative even after postoperative tumor recurrence, suggesting that ctDNA surveillance for postoperative recurrence of gastric cancer may not be appropriate for patients with ctDNA non-shedders.^[24]^ One of the obstacles to the clinical use of ctDNA is the cost of testing, especially with next-generation sequencing and whole-genome sequencing. Post-operative detection of tumor recurrence in gastric cancer with ctDNA shedding facilitates improved cost-effectiveness. Identifying the type of gastric cancer that has ctDNA shedding facilitates the selection of suitable gastric cancer patients to be monitored for recurrence after gastric cancer surgery. Yang study found that worse clinical stage, worse N stage, worse T stage, or cardiac gastric cancer had higher preoperative ctDNA shedding. In addition, only the worse T stage had significantly higher preoperative ctDNA shedding in the multivariate analysis.^[22]^ Leal study then showed that ctDNA was more likely to be detected preoperatively in patients with intestinal subtypes than in patients with diffuse subtypes.^[23]^ Cabel study indicated that ctDNA shedders were not associated with Lauren classification, and in short, different studies have shown inconsistent or even opposite results for pre-operative ctDNA shedding in gastric cancer.^[25]^ In addition, the published studies had small sample sizes, so further large multicentre studies are needed to identify which types of gastric cancer have preoperative ctDNA shedding.

Many studies have explored ctDNA as a predictor of post-operative recurrence in solid tumors, where ctDNA has become a biomarker of tumor recurrence in breast, lung, and colon cancers.^[17–19,21]^ There is currently a small amount of evidence for the use of ctDNA in predicting recurrence of gastric cancer after surgery, and we have shown by meta-analysis that patients with preoperatively positive ctDNA have a higher risk of gastric cancer recurrence after surgery. Inconsistent results were found in the pooled analyses, with Kim study and Yang study showing that although there was a tendency for preoperative ctDNA positivity to be associated with greater tumor recurrence, the results did not reach statistical significance.^[22,24]^ The possible reason for this was that the sample sizes of the 2 studies were small, with Kim study and Yang study having a sample size of 17 and 40, respectively. Leal study then showed that preoperative ctDNA-positive patients had a higher risk of postoperative gastric cancer recurrence.^[23]^ Patients in Leal study received preoperative chemotherapy, and the detection of ctDNA prior to surgery is indicative of a suboptimal response to preoperative chemotherapy in this group.^[23]^ Given that preoperative ctDNA-positive patients have a higher risk of recurrence of gastric cancer, more aggressive surgical treatment and intensive postoperative chemotherapy should be chosen for this group of patients. This pooled analysis on the assessment of preoperative ctDNA to predict the risk of recurrence of gastric cancer included a few studies and a small sample size, and the conclusions still need to be confirmed by a large sample of multicentre studies.

The study showed through a meta-analysis that post-operative ctDNA positive patients have a higher risk of recurrence of gastric cancer after surgery. ctDNA had the following advantages as a liquid biopsy to predict recurrence of gastric cancer after surgery. Firstly, ctDNA predicted recurrence of gastric cancer 6 months earlier than radiographic recurrence and 4 to 8.9 months earlier than clinical recurrence.^[22–24]^ Secondly, the emergence of new tumor genetic variants during and after chemotherapy, especially new driver gene mutations, suggests that ctDNA can detect new tumor genetic variants in time for individualized and precise treatment, and this is another advantage of ctDNA independent of pre-operative tissue genotyping.^[22]^ Serial monitoring of post-operative ctDNA has been shown to increase the sensitivity of detecting tumor recurrence compared to a single post-operative ctDNA test in colon and breast cancer studies.^[19,21,41]^ Yang study also showed that serial monitoring of ctDNA is more likely to detect recurrence of post-operative gastric cancer, so more frequent screening of post-operative ctDNA is beneficial in detecting recurrence of post-operative gastric cancer.^[22]^ In addition, ctDNA was not always positive after surgery for gastric cancer in the postoperative follow-up. Some patients became negative for ctDNA 1 month after surgery and then turned positive again before tumor recurrence, indicating that ctDNA levels and ctDNA dynamics correlated in the postoperative follow-up.^[24]^ However, Cabel study showed that ctDNA did not predict recurrence of gastric cancer after surgery, which is inconsistent with other studies probably because Cabel study tested ctDNA at a low level of 20%, 0% and 8% at baseline preoperatively and postoperatively, respectively. In addition, in Cabel study, ctDNA levels decreased significantly after preoperative FOLFOX-based chemotherapy, so that ctDNA was not detectable in any of the preoperative patients.^[25]^ Similar ctDNA kinetics results were observed in breast and rectal cancers.^[42,43]^ In conclusion, individualized treatment and follow-up plans based on risk factors for recurrence can reduce recurrence and metastasis rates in patients with gastric cancer and improve their survival rates. In addition, ctDNA-guided categorical therapy for colon cancer has reduced adjuvant chemotherapy^[44]^ Future research should look at whether ctDNA can identify recurrence after postoperative chemotherapy for gastric cancer, and whether ctDNA can guide postoperative chemotherapy. As published studies have shown, ctDNA has great potential for clinical application in predicting tumor recurrence and prognosis in operable gastric cancer, and several related studies are already in progress (NCT05029869, NCT03957564, and NCT04576858).

Although the first meta-analysis demonstrated the potential usefulness of ctDNA in predicting tumor recurrence and prognosis in gastric cancer, it is important to acknowledge the following limitations. Firstly, the number of studies and sample size included in this meta-analysis were relatively small. Secondly, the meta-analysis did not include subgroup analysis because of the small number of studies included. Thirdly, there was heterogeneity in the meta-analysis. Fourth, the Suzuki study included in the pooled analysis for RFS included a proportion of patients with colon cancer. Finally, it worth noting that many of the studies included in the prognosis assessment had relatively short follow-up periods, especially for assessing OS, which may not have been long enough to draw definitive conclusions.

## 5. Conclusion

In conclusion, this study showed a higher risk of gastric cancer recurrence in ctDNA-positive patients at presurgery and postsurgery, suggesting that these patients may respond poorly to treatment and should be closely followed. ctDNA could also be used to predict gastric cancer prognosis, with ctDNA-positive patients having a worse OS and a worse RFS.

## Author contributions

**Conceptualization:** Junjie Mi.

**Data curation:** Ruijun Ma.

**Formal analysis:** Junjie Mi, Ruijun Ma.

**Investigation:** Huiying Li.

**Methodology:** Rong Wang.

**Software:** Xiaofang Han.

## Supplementary Material






















