# Supplementary material for: Circulating tumor DNA predicts recurrence and assesses prognosis in operable gastric cancer: A systematic review and meta-analysis
Source: Medicine (Baltimore). 2023 Dec 1;102(48):e36228. doi: 10.1097/MD.0000000000036228 (PMC10695564; doi:10.1097/MD.0000000000036228)
Supplement: Supplementary file 3 [file medi-102-e36228-s003.docx]

**Supplementary appendix 3.**

**Search details**

Two authors independently conducted a comprehensive and systematic search of PubMed, Embase, Scopus, and Web of Science, respectively, up to April 7, 2023.

**PubMed**

#1: Circulating Tumor DNA 8,906

#2: ctDNA 5,272

#3: (Circulating Tumor) AND (DNA) 11,807

#4: (Tumor DNA) AND (Circulating) 11,807

#5: Cell Free Tumor DNA 21,024

#6: (Cell-Free Tumor) AND (DNA) 5,842

#7: (Tumor DNA) AND (Cell-Free) 5,842

#8: #1 or #2 or #3 or #4 or #5 or #6 or #7

((((((Circulating Tumor DNA) OR (ctDNA)) OR ((Circulating Tumor) AND (DNA))) OR ((Tumor DNA) AND (Circulating))) OR (Cell Free Tumor DNA)) OR ((Cell-Free Tumor) AND (DNA))) OR ((Tumor DNA) AND (Cell-Free)) 26,876

#9: gastric tumor 145,396

#10: stomach tumor 129,873

#11: stomach cancer 132,978

#12: Stomach Neoplasms 125,092

#13: Gastric Cancer 162,271

#14: #9 or #10 or #11 or #12 or #13

((((Gastric Cancer) OR (Stomach Neoplasms)) OR (stomach cancer)) OR (stomach tumor)) OR (gastric tumor) 173,609

#15: recurrence 808,076

#16: minimal residual disease 30,531

#17: prognosis 2,190,785

#18: molecular residual disease 7,897

#19: neoplasm recurrence 341,292

#20: #15 or #16 or #17 or #18 or #19

((((Prognosis) OR (molecular residual disease)) OR (minimal residual disease)) OR (neoplasm recurrence)) OR (recurrence) 2,747,864

#21: #8 and #14 and #20

((((((Gastric Cancer) OR (Stomach Neoplasms)) OR (stomach cancer)) OR (stomach tumor)) OR (gastric tumor)) AND (((((((Circulating Tumor DNA) OR (ctDNA)) OR ((Circulating Tumor) AND (DNA))) OR ((Tumor DNA) AND (Circulating))) OR (Cell Free Tumor DNA)) OR ((Cell-Free Tumor) AND (DNA))) OR ((Tumor DNA) AND (Cell-Free)))) AND (((((Prognosis) OR (molecular residual disease)) OR (minimal residual disease)) OR (neoplasm recurrence)) OR (recurrence)) 277

**Embase**

#1: circulating AND tumor AND dna 23,645

#2: ctdna 12,515

#3: 'cell free' AND tumor AND dna 9,986

#4: cell AND free AND tumor AND dna 44,858

#5: #1 or #2 or #3 or #4

circulating AND ('tumor'/exp OR tumor) AND ('dna'/exp OR dna) OR 'ctdna'/exp OR ctdna OR ('cell free' AND ('tumor'/exp OR tumor) AND ('dna'/exp OR dna)) OR (('cell'/exp OR cell) AND free AND ('tumor'/exp OR tumor) AND ('dna'/exp OR dna)) 64,218

#6: gastric tumor 247,303

#7: stomach tumor 263,940

#8: stomach cancer 237,636

#9: Stomach Neoplasms 258,728

#10: Gastric Cancer 217,406

#11: #6 or #7 or #8 or #9 or #10

'gastric tumor'/exp OR 'gastric tumor' OR (gastric AND ('tumor'/exp OR tumor)) OR 'stomach tumor'/exp OR 'stomach tumor' OR (('stomach'/exp OR stomach) AND ('tumor'/exp OR tumor)) OR 'stomach cancer'/exp OR 'stomach cancer' OR (('stomach'/exp OR stomach) AND ('cancer'/exp OR cancer)) OR 'stomach neoplasms'/exp OR 'stomach neoplasms' OR (('stomach'/exp OR stomach) AND ('neoplasms'/exp OR neoplasms)) OR 'gastric cancer'/exp OR 'gastric cancer' OR (gastric AND ('cancer'/exp OR cancer)) 307,543

#12: recurrence 892,999

#13: minimal AND residual AND disease 50,584

#14: 'molecular residual disease' OR (molecular AND residual AND ('disease'/exp OR disease)) 24,738

#15: 'neoplasm recurrence' OR (('neoplasm'/exp OR neoplasm) AND ('recurrence'/exp OR recurrence)) 572,574

#16: 'prognosis'/exp OR prognosis 1,199,919

#17: #12 or #13 or #14 or #15 or #16

'recurrence'/exp OR recurrence OR (minimal AND residual AND ('disease'/exp OR disease)) OR 'molecular residual disease' OR (molecular AND residual AND ('disease'/exp OR disease)) OR 'neoplasm recurrence' OR (('neoplasm'/exp OR neoplasm) AND ('recurrence'/exp OR recurrence)) OR 'prognosis'/exp OR prognosis 1,972,458

#18: #5 and #11 and #17

(circulating AND ('tumor'/exp OR tumor) AND ('dna'/exp OR dna) OR 'ctdna'/exp OR ctdna OR ('cell free' AND ('tumor'/exp OR tumor) AND ('dna'/exp OR dna)) OR (('cell'/exp OR cell) AND free AND ('tumor'/exp OR tumor) AND ('dna'/exp OR dna))) AND ('gastric tumor'/exp OR 'gastric tumor' OR (gastric AND ('tumor'/exp OR tumor)) OR 'stomach tumor'/exp OR 'stomach tumor' OR (('stomach'/exp OR stomach) AND ('tumor'/exp OR tumor)) OR 'stomach cancer'/exp OR 'stomach cancer' OR (('stomach'/exp OR stomach) AND ('cancer'/exp OR cancer)) OR 'stomach neoplasms'/exp OR 'stomach neoplasms' OR (('stomach'/exp OR stomach) AND ('neoplasms'/exp OR neoplasms)) OR 'gastric cancer'/exp OR 'gastric cancer' OR (gastric AND ('cancer'/exp OR cancer))) AND ('recurrence'/exp OR recurrence OR (minimal AND residual AND ('disease'/exp OR disease)) OR 'molecular residual disease' OR (molecular AND residual AND ('disease'/exp OR disease)) OR 'neoplasm recurrence' OR (('neoplasm'/exp OR neoplasm) AND ('recurrence'/exp OR recurrence)) OR 'prognosis'/exp OR prognosis) 930

**Web of science**

#1: Circulating Tumor DNA (All Fields) or ctDNA (All Fields) or (Circulating Tumor) AND (DNA) (All Fields) or (Tumor DNA) AND (Circulating) (All Fields) or Cell Free Tumor DNA (All Fields) or (Cell-Free Tumor) AND (DNA) (All Fields) or (Tumor DNA) AND (Cell-Free) (All Fields) 25,448

#2: gastric tumor (All Fields) or stomach tumor (All Fields) or stomach cancer (All Fields) or Stomach Neoplasms (All Fields) or Gastric Cancer (All Fields) 157,874

#3: recurrence (All Fields) or minimal residual disease (All Fields) or prognosis (All Fields) or molecular residual disease (All Fields) or neoplasm recurrence (All Fields) 895,106

#4: #1 and #2 and # 3 290

**Scopus**

#1: (TITLE-ABS-KEY (circulating AND tumor AND dna) OR TITLE-ABS-KEY (ctdna ) OR TITLE-ABS-KEY ( ( circulating AND tumor ) AND ( dna ) ) OR TITLE-ABS-KEY ( ( tumor AND dna ) AND ( circulating ) ) OR TITLE-ABS-KEY ( cell AND free AND tumor AND dna ) OR TITLE-ABS-KEY ( ( cell-free AND tumor ) AND ( dna ) ) OR TITLE-ABS-KEY ( ( tumor AND dna ) AND ( cell-free ) ) ) 40,531

#2: (TITLE-ABS-KEY (gastric AND tumor) OR TITLE-ABS-KEY ( stomach AND tumor ) OR TITLE-ABS-KEY ( stomach AND cancer ) OR TITLE-ABS-KEY ( stomach AND neoplasms ) OR TITLE-ABS-KEY ( gastric AND cancer ) ) 245,172

#3: (TITLE-ABS-KEY (recurrence) OR TITLE-ABS-KEY (minimal AND residual AND disease) OR TITLE-ABS-KEY (prognosis) OR TITLE-ABS-KEY (molecular AND residual AND disease) OR TITLE-ABS-KEY ( neoplasm AND recurrence ) ) 1,820,006

#4: #1and #2 and #3 ( ( TITLE-ABS-KEY ( circulating AND tumor AND dna ) OR TITLE-ABS-KEY ( ctdna ) OR TITLE-ABS-KEY ( ( circulating AND tumor ) AND ( dna ) ) OR TITLE-ABS-KEY ( ( tumor AND dna ) AND ( circulating ) ) OR TITLE-ABS-KEY ( cell AND free AND tumor AND dna ) OR TITLE-ABS-KEY ( ( cell-free AND tumor ) AND ( dna ) ) OR TITLE-ABS-KEY ( ( tumor AND dna ) AND ( cell-free ) ) ) ) AND ( ( TITLE-ABS-KEY ( gastric AND tumor ) OR TITLE-ABS-KEY ( stomach AND tumor ) OR TITLE-ABS-KEY ( stomach AND cancer) OR TITLE-ABS-KEY ( stomach AND neoplasms ) OR TITLE-ABS-KEY ( gastric AND cancer ) ) ) AND ( ( TITLE-ABS-KEY (recurrence) OR TITLE-ABS-KEY ( minimal AND residual AND disease ) OR TITLE-ABS-KEY ( prognosis ) OR TITLE-ABS-KEY ( molecular AND residual AND disease ) OR TITLE-ABS-KEY ( neoplasm AND recurrence ) ) ) 829

#5: #4 and (Article or Conference Paper)

( ( TITLE-ABS-KEY ( circulating AND tumor AND dna ) OR TITLE-ABS-KEY ( ctdna ) OR TITLE-ABS-KEY ( ( circulating AND tumor ) AND ( dna ) ) OR TITLE-ABS-KEY ( ( tumor AND dna ) AND ( circulating ) ) OR TITLE-ABS-KEY ( cell AND free AND tumor AND dna ) OR TITLE-ABS-KEY ( ( cell-free AND tumor ) AND ( dna ) ) OR TITLE-ABS-KEY ( ( tumor AND dna ) AND ( cell-free ) ) ) ) AND ( ( TITLE-ABS-KEY ( gastric AND tumor ) OR TITLE-ABS-KEY ( stomach AND tumor ) OR TITLE-ABS-KEY ( stomach AND cancer ) OR TITLE-ABS-KEY ( stomach AND neoplasms ) OR TITLE-ABS-KEY ( gastric AND cancer ) ) ) AND ( ( TITLE-ABS-KEY ( recurrence ) OR TITLE-ABS-KEY ( minimal AND residual AND disease ) OR TITLE-ABS-KEY ( prognosis ) OR TITLE-ABS-KEY ( molecular AND residual AND disease ) OR TITLE-ABS-KEY ( neoplasm AND recurrence ) ) ) AND ( LIMIT-TO ( DOCTYPE , "ar" ) OR LIMIT-TO ( DOCTYPE , "cp" ) ) 561
