# Supplementary material for: Circulating tumor DNA predicts recurrence and assesses prognosis in operable gastric cancer: A systematic review and meta-analysis
Source: Medicine (Baltimore). 2023 Dec 1;102(48):e36228. doi: 10.1097/MD.0000000000036228 (PMC10695564; doi:10.1097/MD.0000000000036228)
Supplement: Supplementary file 4 [file medi-102-e36228-s004.docx]

**Supplementary appendix 4.**

ctDNA testing time, definition of the positive ctDNA and treatment strategy in the studies included in the systematic review and meta-analysis

| Author | Year | ctDNA testing time | Definition of positive ctDNA | Treatment strategy |
| --- | --- | --- | --- | --- |
| Kim | 2019 | Presurgery  Postsurgery | Any cancer-specific rearranged sequence detected in any postoperative plasma sample is considered positive for ctDNA. | Patients underwent curable surgical resection followed by adjuvant chemotherapy in some patients. |
| Cabel | 2019 | Baseline  Presurgery  Postsurgery | Positive ctDNA was defined as a pathogenic variant in a driver gene with an MAF greater than 1% in the tumor. | Preoperative chemotherapy consisted of six cycles of 5-fluorouracil and oxaliplatin, and some patients received nab-paclitaxel as well as trastuzumab. |
| Yang | 2020 | Presurgery  Postsurgery | The detection of one or more mutations. | Eligible patients underwent gastrectomy with curative intent, followed by adjuvant chemotherapy. |
| Leal | 2020 | Baseline  Presurgery  Postsurgery | Each mutation that was identified only by cfDNA sequencing. | Patients were randomized to receive three preoperative 21-day cycles of intravenous epirubicin, cisplatin or oxaliplatin, and oral capecitabine followed by three postoperative cycles of intravenous epirubicin, cisplatin or oxaliplatin, and oral capecitabine or to receive the same preoperative regimen followed by postoperative radiotherapy combined with daily capecitabine and weekly cisplatin. |
| Suzuki | 2020 | Presurgery  Postsurgery | A mutation was found with the highest frequency in the patient and a 1% frequency in the gene. | Not available |
| Fedyanin | 2020 | Presurgery  Postsurgery | The content of ctDNA was more than 0.5 copies of mutant DNA per milliliter of plasma. | Some patients received adjuvant or perioperative chemotherapy. |
| Huffman | 2022 | Presurgery  Postsurgery | Samples with at least two tumor-specific variants were defined as ctDNA-positive. | All patients received treatment and follow-up in accordance with standard clinical practice and at the investigator’s discretion. |
| Yuan | 2022 | Presurgery  Postsurgery | One or more variants detected in the plasma were present in at least 2% of the primary tumors. | All the patients received curative-intent standard-of-care therapy. |

**Abbreviation**: MAF, mutant allelic frequency.
