# Supplementary material for: Circulating tumor DNA predicts recurrence and assesses prognosis in operable gastric cancer: A systematic review and meta-analysis
Source: Medicine (Baltimore). 2023 Dec 1;102(48):e36228. doi: 10.1097/MD.0000000000036228 (PMC10695564; doi:10.1097/MD.0000000000036228)
Supplement: Supplementary file 5 [file medi-102-e36228-s005.docx]

**Supplementary appendix 5.**

P-value of preoperative ctDNA shedding in gastric cancer patients

| Author | Year | T stage | N stage | Localization | Pathological  response | Degree of differentiation | clinical stage | Lauren classification |
| --- | --- | --- | --- | --- | --- | --- | --- | --- |
| Kim | 2019 | 0.32 | >0.05 | NA | NA | NA | >0.05 | >0.05 |
| Cabel | 2019 | 0.77 | 1 | 0.54 | 0.18 | NA | NA | 1 |
| Yang | 2020 | 0.01 | 0.03 | 0.03 | NA | 0.15 | <0.01 | 0.57 |
| Leal | 2020 | NA | NA | NA | NA | 0.07 | NA | 0.02 |
| Suzuki | 2020 | NA | NA | NA | NA | NA | NA | NA |
| Fedyanin | 2020 | NA | NA | NA | NA | NA | NA | NA |
| Huffman | 2022 | NA | NA | NA | NA | NA | NA | NA |
| Yuan | 2022 | NA | NA | NA | NA | NA | NA | NA |
