# Supplementary material for: Circulating tumor DNA predicts recurrence and assesses prognosis in operable gastric cancer: A systematic review and meta-analysis
Source: Medicine (Baltimore). 2023 Dec 1;102(48):e36228. doi: 10.1097/MD.0000000000036228 (PMC10695564; doi:10.1097/MD.0000000000036228)
Supplement: Supplementary file 8 [file medi-102-e36228-s008.docx]

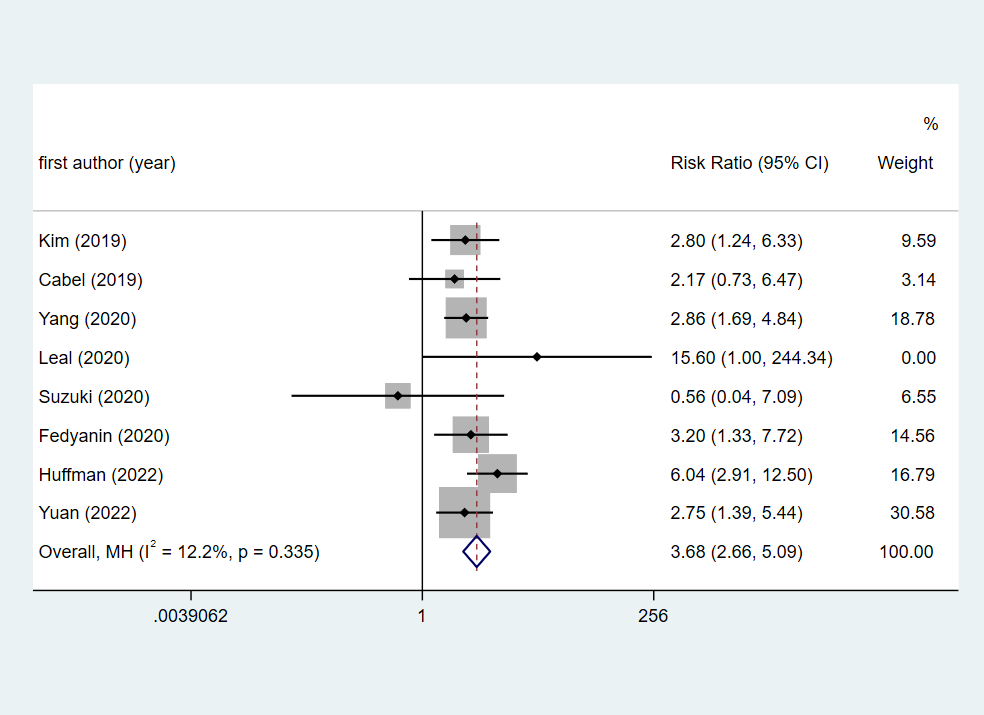


**Supplementary appendix 8.**

A forest plot of the pooled effect for relative risk in postsurgical ctDNA prediction of gastric cancer recurrence after surgery using fixed effects model.
