# Supplementary figures and images for: Circulating tumor DNA predicts recurrence and assesses prognosis in operable gastric cancer: A systematic review and meta-analysis
Source: Medicine (Baltimore). 2023 Dec 1;102(48):e36228. doi: 10.1097/MD.0000000000036228 (PMC10695564; doi:10.1097/MD.0000000000036228)

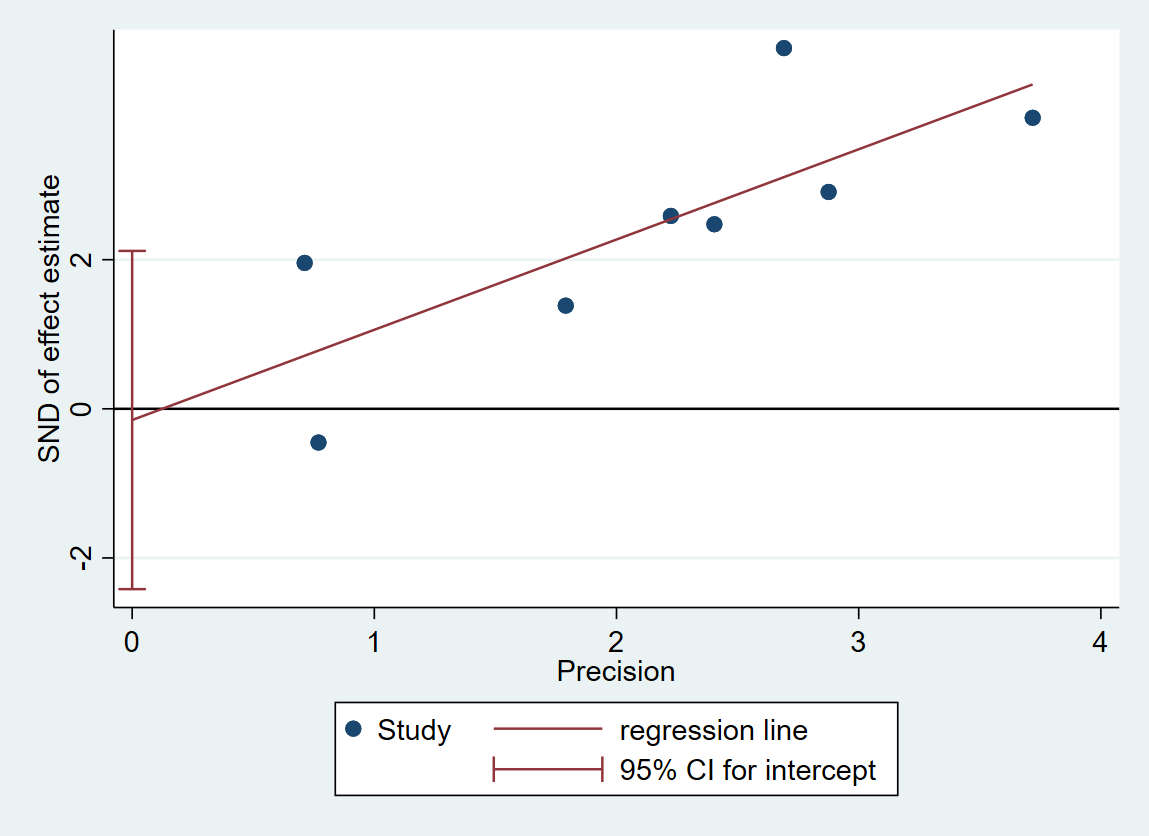


**Supplementary appendix 9.**

The Egger test for ctDNA prediction of gastric cancer recurrence.

Supplement: Supplementary file 9 [file medi-102-e36228-s009.docx]

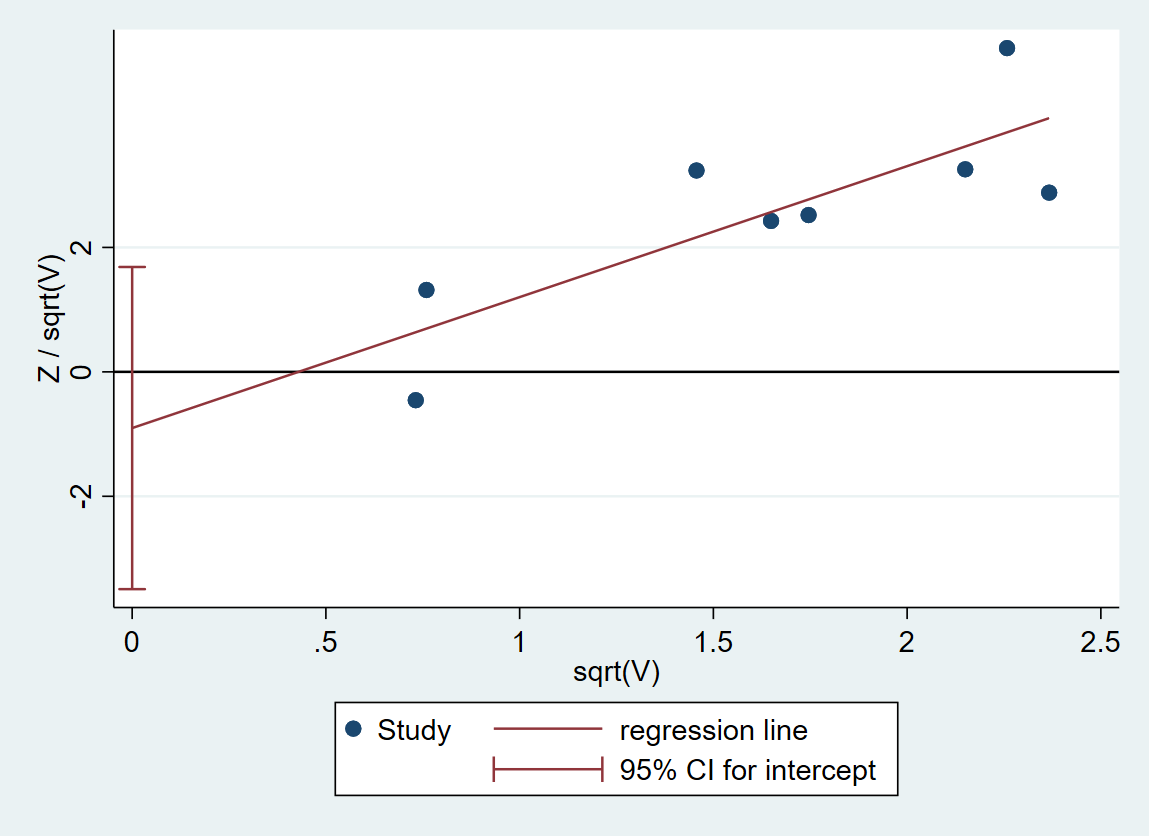


**Supplementary appendix 10.**

The Harbord test for ctDNA prediction of gastric cancer recurrence.

Supplement: Supplementary file 10 [file medi-102-e36228-s010.docx]
